# Multiple Sclerosis in Pregnancy and its Role in Female Fertility: A Systematic Review

**DOI:** 10.5935/1518-0557.20210022

**Published:** 2021

**Authors:** Rivia Lamaita, Carolina Melo, Cláudia Laranjeira, Paula Barquero, Joyce Gomes, Agnaldo Silva-Filho

**Affiliations:** 1 Department of Gynecology and Obstetrics of the School of Medicine of the Federal University of Minas Gerais, Belo Horizonte, Minas Gerais, Brazil; 2 Rede Mater Dei de Saúde, Belo Horizonte, Minas Gerais, Brazil; 3 Merck Brazil

**Keywords:** multiple sclerosis, pregnancy, fertility, systematic review

## Abstract

Multiple sclerosis (MS) is a neurological disease that typically affects young women of reproductive age. There are still many questions and heterogeneous clinical approaches partly due to the lack of consensus and guidelines. For many years, women with MS have been discouraged from getting pregnant for fears that the disease might negatively affect the fetus or increase their obstetric risk or for claims that the disease might decrease fertility. However, fertility does not seem to be impaired to a larger extent in women with MS. Therefore, all healthcare providers involved in the follow-up of multiple sclerosis patients must be prepared to discuss future fertility, pregnancy, and others matters, in addition to providing them with the best possible counseling. This study presents data based on updated evidence and discusses fertility and pregnancy in patients with MS with respect to the impacts of pregnancy on the risk and prognostic factors tied to MS, and the impact of MS on pregnancy outcomes and fertility treatments administered to females with MS. In conclusion, a clear relationship between infertility and MS has not been established. There seems to exist a link between disease aggressiveness and progression with several processes that might impair fertility. However, MS does not stand as a contraindication to assisted reproductive technology. From the several studies analyzed, it is possible to conclude that pregnancy is possible in women with MS. It is important to discuss and plan the ideal moment to start treatment and managing pregnancy and contraception aiming at better results.

## INTRODUCTION

Multiple sclerosis (MS) is a neurological disease characterized by motor, sensory and/or cognitive impairment interspersed with episodes of remission, triggered by an abnormal demyelinating immune response in the central nervous system and spinal cord. It affects people aged 30-40 years on average. The prevalence ratio of multiple sclerosis from women to men has increased sharply in recent decades (2.3 to 3.5:1), which indicates a significant increase in multiple sclerosis among women (Harbo *et al*., 2013). In Brazil, prevalence ranges from 5 to 20 individuals per 100,000 inhabitants (Marques *et al*., 2018), among which are women of childbearing age. A retrospective analysis conducted in the public healthy registry between 2000 and 2015 found a total of 28,401 MS patients treated in the country. The majority were females (73.3%) with a mean age of 36.8 years living in the Brazilian Southeast (58.9%) (Maia Diniz *et al*., 2018).

The development of MS is related to genetic factors, such as the presence of HLA allotypes (HLA-DR2), in association with environmental factors such as sun exposure, prolonged low vitamin D levels, smoking, and latent infection by some types of Epstein Barr virus. When associated, triggering factors generate a cross-autoimmune response with myelin proteins, triggering the destruction of oligodendrocytes, axonal damage, perivascular inflammation, and chemical changes in myelin lipid constituents. The lesions form atrophic plaques containing fibrosis, which are mainly found in the lateral and posterior columns of the spinal cord, brain white matter, optic nerves, and periventricular areas. The characteristics associated with the location of each plaque correlate with the seriousness and clinical presentation (subtype) of the disease (Salapa *et al*., 2017).

For many years, women with MS have been discouraged from getting pregnant for fears of not being able to care for their children due to fatigue or disability, or because of concerns around passing on the genetic susceptibility to an autoimmune condition to their offspring. There is no clear data to indicate that women with MS suffer from impaired fertility, although a recent study suggested that patients with MS had decreases in ovarian reserve. General infertility rates revolve around 10-20% in couples from western countries, with infertility in women with MS potentially accounting as a co-occurrence (Thöne *et al*., 2015).

However, studies in recent decades have shown a protective effect of pregnancy in the course of MS, with reduced outbreak rates due to the immunological complacent gestational state reaching up to 70% during the third trimester, with return of episodes only in the puerperium (Amato *et al*., 2017).

Patients with MS may also be faced with infertility. A significantly greater proportion of women with MS has been handed a diagnosis of infertility when compared to women without MS (8.5% *vs*. 8.1%; *p*=0.0006). When stratified by age, a greater proportion of women with MS aged 18 to 34 and aged 42 years and older have been diagnosed with infertility compared to women without MS (Houtchens *et al*., 2020). The few studies on the subject present conflicting evidence about whether fertility in women with MS might be reduced and whether the use of infertility treatment affects the course of MS. Assisted reproductive technology has been reported to increase risk, although not enough studies support such statement (Xie *et al*., 2020).

*In vitro* studies have linked activated estrogen receptors in genetic transcription to the activation of anti-inflammatory cytokines and the pathway of cholesterol metabolism, which are important elements in the remyelination of oligodendrocytes during pregnancy. The activation of these receptors favors the control of the disease mainly in the third trimester, when high levels of estrogen and progesterone are present. Similarly, it is already known that estrogen is a global neuroprotective agent that favors adequate neurocognitive development and expression (Voskuhl *et al*., 2019).

Thus, it is advantageous that pregnancy has immunomodulatory effects capable of controlling the disease, while estrogen provides for fetal neurological protection and development in mothers with the disease (Voskuhl & Momtazee, 2017). Safe medications are available for use during pregnancy and breastfeeding, including corticosteroids, some Immunomodulators, and drugs to treat symptoms such as muscle relaxants and antidepressants (Amato *et al*., 2017). However, family planning and counseling are of paramount importance in the management of the disease. MS must be well controlled before conception, since some adaptation and adjustments to medication are required before pregnancy or the introduction of infertility treatments (Ciccarelli, 2019).

Controversy still looms over fertility along the course of the disease. The mechanisms associating MS and the hypothalamic-pituitary-ovary axis and how they interfere with the various stages of reproductive life have not been fully elucidated (Sepúlveda *et al*., 2016).

Thus, the present study aimed to collect data based on current evidence and discuss fertility and pregnancy in patients with MS with respect to the impacts of pregnancy on the risk and prognosis of MS, and the potential impacts of MS on pregnancy outcomes and treatments used to restore fertility in women with MS.

## METHODS

This review was written in accordance with the PRISMA protocol (Liberati *et al*., 2009). Searches for papers were performed in the Scielo, PubMed and Lilacs databases in February 2020, and included studies conducted in the last five years with papers written in English. The references cited in the selected papers were analyzed for additional studies that had not been identified during the search for papers.

**Figure 1:**
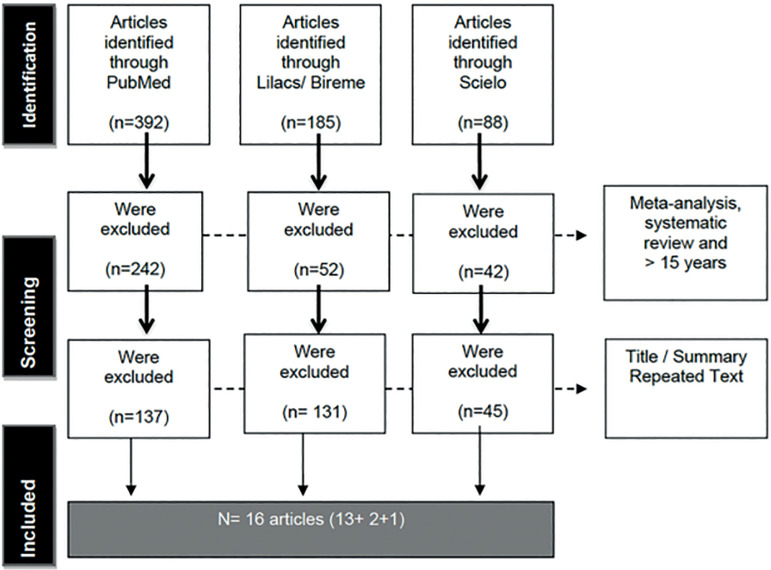
Flowchart showing article selection phases.

The expression used in the searches combined the following terms present in the Medical Subject Headings (MeSH) and its correspondents in the Descriptors in Health Sciences (DeCS): (*Multiple sclerosis*) AND (*Pregnancy*) AND (*Fertility*). The following were included in this review: cohort case studies; case reports; cross-sectional, experimental, and pilot studies; and randomized clinical trials (Figure 1).

## RESULTS

A total of 16 papers were found, of which 15 were observational studies and one was a randomized clinical trial. They were published between 2008 and 2020. Study bias was evaluated based on the *Newcastle-Ottawa Scale* and Jadad scales (Figure 1).

A total of 665 records were found in the literature search, from which 336 meta-analyses, systematic reviews, and articles published for more than 15 years were excluded (Figure 1). In a second review of the remaining articles, another 313 were excluded for presenting repeated text in the title and abstract and for not contemplating the objectives set out for our review. Sixteen articles remained in the end and were included in this review. The included studies were categorized as follows based on their characteristics (Table 1).

**Table 1:** Overall characteristics of selected articles.

Study	Type of study	Population (N)	Outcome	Score Checklist Downs and Black
**Impact of pregnancy on MS risk and prognosis**				
^Altintas *et al*., 2015^	Retrospective cohort	199	Women with MS can get pregnant safely, and the condition does not impair the functional capacity of women with MS during or after pregnancy, although fertility is reduced even before diagnosis. Pregnancy has a favorable effect on the transition from progressive to remittent forms of the disease.	17/27
^Fares *et al.*, 2016^	Prospective cohort	29	MS does not increase gestational risk and usually triggers remission of the disease for up to 2 years after delivery, although in the long run symptoms seen prior to pregnancy tend to reemerge.	18/27
^Ferraro *et al*., 2017^	Case-control	803	MS does not affect the rates of deliveries from C-sections or breastfeeding. 16% of women with MS and no children report fear of physical incapacity to care, fear of interrupting treatment during pregnancy and the disease getting worse, and especially fear of transmitting the disease to their offspring.	15/27
^Spadaro *et al*., 2019^	Case-control	134	Deciduous immune cells at the maternal-fetal interface modulate systemic immune function by decreasing cellular action, except for M2 monocytes, which control the disease during pregnancy.	20/27
^Zuluaga *et al*., 2019^	Prospective cohort	501	Although hormonal changes in the various hypothalamic-pituitary axes affect some autoimmune diseases, pregnancy does not change the prognosis of patients with MS.	19/27
**Impact of MS on pregnancy outcomes**				
^Houtchens *et al.*, 2018^	Retrospective cohort	2115	Pregnant women with MS have more preterm labor, peripartum infection, and hereditary congenital malformations, although they present other associated comorbidities and are older on average compared to individuals without MS, which makes it difficult to analyze isolated data.	16/27
Moberg *et al*., 2020	Retrospective cohort	4692	Women with MS have reportedly fewer children, primarily because a diagnosis of MS negatively affects the decision to have children. MS alone does not affect the percentage of miscarriages or number of ectopic pregnancies.	19/27
Winkelmann *et al*., 2019	Randomized clinical trial	70	Treatment with glatiramer acetate or interferon does not increase the risk of spontaneous miscarriage or birth defects during the first trimester of pregnancy.	20/27
**Impact of MS on fertility and infertility treatments**				
Correale *et al*., 2012	Prospective cohort	16	ART was associated with a 7-fold increase in the risk of MS exacerbation and with a 9-fold increase in the risk of enhanced disease activity as seen in MRI scans of lesions. Worsening was associated with higher number of cells producing IL-8, IL-12, IFNc, and TGF-b, as well as increased VEGF production by CD4. T cells and CXCL-12 plasma levels, all GnRH-mediated.	19/27
Ghafoori *et al*., 2020	Cross-sectional	25	Iranian women with MS avoided pregnancy for fear of motor impairment affecting their ability to care for their children, fear of sclerosis medications affecting the menstrual cycle or limitation of techniques and reproduction treatment due to the presence of the disease.	17/27
Hellwig *et al*., 2009	Retrospective cohort	23	The results confirm an increased relapse rate after as many as 78 ART cycles in women with MS. They indicate that women with MS should be informed that there is a possible risk of increased RR.	19/27
Michel *et al*., 2012	Retrospective cohort	32	A significant increase in the annualized relapse rate (ARR) was observed during the 3 months following IVF compared with the same period just before IVF. The significant increase in relapses was associated with the use of GnRH agonists as well as IVF failure.	19/27
Roux *et al*., 2015	Retrospective cohort	115	There is no direct impact of MS on fertility and treatment does not interfere with the mean age of spontaneous pregnancy.	17/27
Sepúlveda *et al*., 2016	Cross-sectional	25	There was no difference between levels of FSH, LH, inhibits, estrogen, progesterone, free testosterone, anti-Müllerian hormone (AMH), or ovarian reserve in women with controlled MS or without the disease. However, patients with uncontrolled disease and subjects with active MS had smaller ovaries with fewer follicles and decreased AMH levels.	18/27
Smith *et al*., 2019	Prospective cohort	63	Family planning has to be considered since there is no direct relationship between decreased fertility and MS. Women who discontinue treatment after finding out they were pregnancy had more frequent and intense postpartum episodes.	17/27
Thöne *et al*., 2015	Cross-sectional	148	MS and other localized autoimmune diseases are related to lower levels of AMH during activation, and increased endoglin (endothelial transmembrane receptor) unrelated to IL-B (autoimmune lymphocytic signaling). There is no relationship between the production of anti-ovarian antibodies and MS.	19/27

### The impact of pregnancy on MS risk and prognosis

There were two prospective cohorts, two control-cases and one retrospective cohort that looked into the potential impacts of pregnancy on MS risk and prognosis. The results showed that women with MS can get pregnant safely, and that the condition does not impair the functional capacity of women with MS during or after pregnancy. Pregnancy has a favorable effect on the transition from progressive to remittent forms of the disease, and MS does not increase gestational risks; pregnancy usually triggers remission of the disease for up to two years after delivery, although in the long run symptoms seen prior to pregnancy tend to reemerge. There is no prenatal test to determine the risk of transmitting MS, and about 80% of the patients have a negative family history for the disease. Overall, the risk of a patient with MS having a child with the disease revolves around 2-3%, which is a chance 20 times greater than that of the general population in Western countries presenting the disease.

### The impact of MS on pregnancy outcomes

Two retrospective cohorts and one randomized clinical trial were included. The fertility of patients with sclerosis does not seem to be affected by the evolution of the disease or approved treatments, and there is no increased risk of spontaneous miscarriage, C-section, or number of ectopic pregnancies. However, these patients have more preterm labor, peripartum infection, and hereditary congenital malformations, although they present other associated comorbidities and are older on average compared to individuals without MS, which makes it difficult to analyze isolated data. Women with MS have reportedly fewer children, primarily because a diagnosis of MS negatively affects the decision to have children.

### The impact of MS on fertility and infertility treatments

Two prospective cohorts, three retrospective cohorts and three cross-sectional studies were included in this review. Studies reporting increases in symptoms in women undergoing unsuccessful assisted reproductive technology (ART) treatments enrolled few patients and did not allow for significant data analysis. ART was associated with a 7-fold increase in the risk of MS exacerbation and with a 9-fold increase in the risk of enhanced disease activity as seen in MRI scans of lesions in patients prescribed the pituitary block protocol with a GnRH analog, and not when the block was performed with antagonists. There were no differences regarding injectable gonadotropins used to induce ovulation. Significant increases in relapsing disease were associated with the use of GnRH agonists and IVF failure. The proposed mechanisms included: cessation of DMT, stressful events associated with infertility, and immunological changes induced by GnRH, as well as augmented immune cell migration across the blood-brain barrier.

There was no difference between levels of FSH, LH, inhibits, estrogen, progesterone, free testosterone, anti-Müllerian hormone (AMH), or ovarian reserve in women with controlled MS or without the disease. However, patients with uncontrolled disease and subjects with active MS had smaller ovaries with fewer follicles and decreased AMH levels. No relationship was observed between MS and the production of anti-ovarian antibodies.

Despite the small sample size and the limited number of studies examining the subject, the data suggested that the use of assisted reproductive technology is not contraindicated in patients with MS; instead, it is recommended that ART be performed during a stable period of the disease and that treatment be maintained until pregnancy has been confirmed.

## DISCUSSION

Pregnancy in patients with MS and its implications in the eyes of physicians and individuals with the disease have been scarcely discussed, although three quarters of the patients with MS are women of childbearing age and at least a fifth of them will eventually have children after the onset of the disease. Proper management of patients in this situation requires multidisciplinary knowledge and study (Houtchens *et al*., 2018).

The discussion about the possibility of women with MS enjoying safe pregnancies started with an observational multicenter study (PRIMS) conducted in Europe in 1998, which described a reduction in the rates of disease recurrence during pregnancy, especially in the third trimester (Confavreux *et al*., 1998). Meanwhile, disease-modifying therapies (DMT) emerged in the 2000s to control the disease and improve the quality of life of women with MS, allowing some to consider the possibility of becoming pregnant.

Estrogen, human chorionic gonadotropin (HCG), and progesterone trigger changes in the lymphocytic response profile. They suppress the T-Helper 1 (TH1) response, thereby reducing the release of interferon gamma and tumor necrosis factor alpha associated with MS episodes, in addition to increasing the TH2 response related to the circulation of interleukins 4 and 10, involved in maternal immune tolerance toward the presence of the fetus, thereby reducing the intensity and number of episodes of MS during pregnancy (Fragoso *et al*., 2018).

It is well established in literature that pregnancy is safe in patients with controlled MS. Evidence suggests that minimizing the frequency of episodes before pregnancy correlates with better outcomes in the immediate postpartum (Pozzilli & Pugliatti, 2015). Currently, although there are several drugs approved for the treatment of multiple sclerosis, only glatiramer acetate or interferon have not increased the risk of spontaneous miscarriage or birth defects during the first trimester of pregnancy and in breastfeeding. However, the modification of the therapeutic regimen should be thought of individually and before conception. Drugs should be chosen based on the history of episodes and disease progression patterns (Coyle, 2016; Winkelmann *et al*., 2019).

Most studies show no significant difference in fecundity and fertile capacity of women with early controlled MS. However, the use of assisted reproductive technology is five times greater among patients with advanced disease (Cavalla *et al*., 2006). Some authors relate the circulation of inflammatory cytokines during episodes with decreases in ovarian reserve and hormonal changes. This fact is directly related to the intensity and activity of the disease, which indicates that patients with adequately controlled MS on therapy since the early stages of the disease may not be affected (Sepúlveda *et al*., 2016). Only one paper described fertility decreases even before diagnosis, without however discussing potential associated causes (Altintas *et al.,* 2015).

The immune response of MS also increases the prevalence of endometriosis through shared mechanisms still unknown. In addition, some drugs used in treatment such as cyclophosphamide and mitoxantrone have deleterious effects on oocytes, decrease the ovarian reserve, and causing amenorrhea in up to 30% of the patients. These events might increase the indication of assisted reproductive technology for affected women (Cavalla *et al*., 2006; Amato *et al*., 2017).

Sexual dysfunction affecting patients with MS related to depression and decreased quality of life should also be considered. Women with MS suffer from loss of sensitivity in the genital region, with ensuing changes in libido and primary impairment of sexual function affecting fertility (Delaney & Donovan, 2017).

Overall, women with MS are less likely to have a live birth, more likely to have a diagnosis of infertility, and less likely to receive infertility treatment compared to women without MS (Houtchens *et al*., 2020).

The low number of pregnancies among patients with MS might be explained by the need to postpone pregnancy as a result of the diagnosis of the disease and the prescription of therapies that include medication with potentially adverse pregnancy-related side effects. The burden of functional and cognitive impairment and their impacts on childcare, the fear of pregnancy triggering the progression of the disease, the impact of the disease on gestational risk and the fetus, and the risk of transmitting the disease to offspring have been described by MS patients as factors that discourage reproduction (Roux *et al*., 2015; Moberg *et al*., 2020; Ghafoori *et al*., 2020; Ferraro *et al.,* 2017).

It is essential to navigate through the prescription of therapy, disease management, and pregnancy timing while engaging in multidisciplinary discussions on family planning (Smith *et al*., 2019). It has been established that the use of hormone therapies (contraceptives, ovarian stimulants, and luteal phase stabilizers) is not related to the onset or exacerbation of the disease. However, the administration of combined hormones for a prolonged period of time should be avoided in patients with low mobility due to increased risk of venous thromboembolism. Likewise, there is no evidence that hormones lower the efficacy of disease modifying therapies (DMTs) or vice versa (Kaisey *et al*., 2018).

## CONCLUSION

From the several studies analyzed, it can be concluded that pregnancy is possible in women with MS. This matter should be discussed with patients with MS, since some avoid getting pregnant for fear of complications and for being unaware of the positive relationship between pregnancy and the disease and the possibility of using drug classes that are harmless for the mother and the fetus. In addition, the use of contraceptives, the ideal point in time to start treatment, and pregnancy preparations must be planned properly so that the best outcomes for the mother and fetus are achieved.

The relationship between infertility and MS is not fully understood. There seems to exist a link between aggressiveness and progression of the disease and several processes that may impair fertility. However, MS does not contraindicate the use of assisted reproductive technology as needed. Further studies should be conducted to better define the interactions between the disease and infertility, along with the best procedures indicated for each stage or group of therapies in use.
